# A Method for Identification of the Methylation Level of CpG Islands From NGS Data

**DOI:** 10.1038/s41598-020-65406-1

**Published:** 2020-05-25

**Authors:** Leonid A. Uroshlev, Eldar T. Abdullaev, Iren R. Umarova, Irina A. Il’icheva, Larisa A. Panchenko, Robert V. Polozov, Fyodor A. Kondrashov, Yury D. Nechipurenko, Sergei L. Grokhovsky

**Affiliations:** 1 Engelhardt Institute of Molecular Biology, Russian Academy of Sciences, Moscow, Russia; 2 Vavilov Institute of General Genetics, Russian Academy of Sciences, Moscow, Russia; 3 Max Planck Institute for Molecular Genetics, Berlin, Germany; 4 Faculty of Computational Mathematics and Cybernetics, Moscow State University, Moscow, Russia; 5 Institute of Theoretical and Experimental Biophysics, Russian Academy of Sciences, Puschino, Russia; 6 Institute of Science and Technology Austria, Klosterneuburg, Austria

**Keywords:** Biophysical chemistry, Chemical modification, DNA, Cancer genomics

## Abstract

In the course of sample preparation for Next Generation Sequencing (NGS), DNA is fragmented by various methods. Fragmentation shows a persistent bias with regard to the cleavage rates of various dinucleotides. With the exception of CpG dinucleotides the previously described biases were consistent with results of the DNA cleavage in solution. Here we computed cleavage rates of all dinucleotides including the methylated CpG and unmethylated CpG dinucleotides using data of the Whole Genome Sequencing datasets of the 1000 Genomes project. We found that the cleavage rate of CpG is significantly higher for the methylated CpG dinucleotides. Using this information, we developed a classifier for distinguishing cancer and healthy tissues based on their CpG islands statuses of the fragmentation. A simple Support Vector Machine classifier based on this algorithm shows an accuracy of 84%. The proposed method allows the detection of epigenetic markers purely based on mechanochemical DNA fragmentation, which can be detected by a simple analysis of the NGS sequencing data.

## Introduction

DNA methylation level of CpG islands, genomic sequences with a high occurrence of methylated CpG dinucleotides, is an important regulator of gene expression. The level of gene expression can increase or decrease, depending on the methylation level in the CpG sites inside a CpG island. Analysis of methylation levels in regulatory regions of various genes can provide information on their involvement in the development of various diseases, including cancer.

Previously we showed that sonication of restriction DNA fragments leads to sugar-phosphate backbone breaks, which depend on the nucleotide sequence. Breaks in CpG dinucleotides occur about 1.5 times more often than in other dinucleotides^1^. Then we analyzed the genomic reads from Next-Generation Sequencing (NGS) data and showed that fragmentation methods based on the action of the hydrodynamic forces on DNA produces a similar bias in the cleavage rates^2^ (relative frequencies of dinucleotide breaks denoted here as a cleavage rates). The only discrepancy is in the cleavage rate of CpG dinucleotides. We assume that this result can be explained by methylation of cytosines^2^. Recently it was shown that in the honeybee genome methylated CpG dinucleotides break more frequently than unmethylated ones^3^.

In this work the cleavage rates for methylated and unmethylated CpG dinucleotides of human genome were estimated. We found that the cleavage rate for methylated CpG dinucleotides is about 1.5 times higher than that for unmethylated ones. On the basis on this observation one can estimate the CpG methylation level in CpG islands without any experimental data on DNA methylation. Further, we show that tumor and healthy tissues differ significantly in the methylation status of CpG islands. In human somatic cells approximately 80% of CpG dinucleotides are methylated. Bisulfite sequencing was the first method for detection of cytosine methylation^4^ (see also^5,6^). However, sample preparation for bisulfite sequencing and subsequent data processing is overly expensive of time. On the basis of our results, the criterion can be developed, by which the NGS data can be used for defining the total level of the CpG methylation in the given cell type without any additional experiments.

## Results

**Figure 1:**
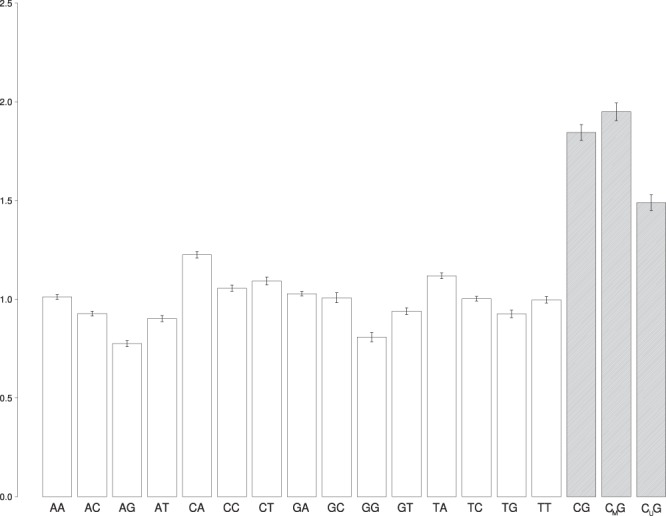
Average cleavage rates r(XY) of 18 dinucleotides (namely, 16 common dinucleotides along with methylated CpGs (C_M_G) and unmethylated CpGs (C_U_G)). Cleavage rates are plotted on Y axis, dinucleotides are listed on X axis. CpG methylation results in a substantial increase of the cleavage rates in comparison with unmethylated CpGs and other dinucleotides.

We randomly selected 100 whole genome sequencing datasets from the 1000 Genomes project that contained reads mapped on a reference genome (GRCh37). According to^7^, these samples were prepared by Covaris ultrasonic DNA shearing. On the basis of the bisulfite-sequencing data obtained from NGSmethDB^8^, we identified the methylation status for all CpG dinucleotides in lymphoblastoid cell line. We filtered only those CpG dinucleotides that were reliably classified as methylated or not (see Methods). On the basis of 5'-read coordinates of each read in each dataset, cleavage rates for all dinucleotides were computed in the following way:

1 $$r(XY)=\frac{n(XY)}{N\ast p(XY)},$$

where *X,Y* = {A, T, G, C_U_ (unmethylated cytosine), C_M_ (methylated cytosine)}; *n*(*XY*) is an overall number of reads in the dataset with the 5′-end nucleotide, in which *X* precedes *Y* in the reference genome; N is an overall number of reads; *p*(*XY*) is an average fraction of *XY* dinucleotides in the genome fragment of length 200 bp around read start (*i*) position^2^. Besides, we computed the average cleavage rates of methylated and unmethylated CpG dinucleotides separately (see Supplementary Tables 3 and 4). As a result, *r*(*XY*) values, where *XY* denotes 16 common dinucleotides + methylated CpGs (C_M_G) and unmethylated CpGs (C_U_G), were calculated on the bases of 100 randomly selected datasets (Fig. 1).

The results of the ANOVA, Kruskal-Wallis test analysis suggest that the effect of the dinucleotide type on the relative frequency of its ultrasonic cleavage is statistically significant at the level p <α = 0.05 (see Supplementary Table 4). The results of the multiple comparisons, Duncantest and Kruskal-Wallis test, are shown in the Tables (see Supplementary Tables 2 and 3). The sample means in the Table 2 Suppl. are combined into subsets so that the means from different subsets (columns 1, 2, 7, 8, 9, 10) differ statistically significantly at the p < 0.05. Therefore the mean of *r*(C_M_G) is the largest of all three values.

According to^9^, tumor cells have a very specific landscape of methylation, especially in apoptosis-related genes. It is noteworthy that CpG methylation is a stochastic process; therefore, only an average methylation of CpG islands matters, not a single dinucleotide status^10^. We can compute the cleavage rates in each CpG island separately and predict if CpG island is hypo- or hypermethylated. Comparison of the CpG methylation levels in CpG islands of specific genes may allow one to distinguish between healthy and cancerous tissues.

**Figure 2:**
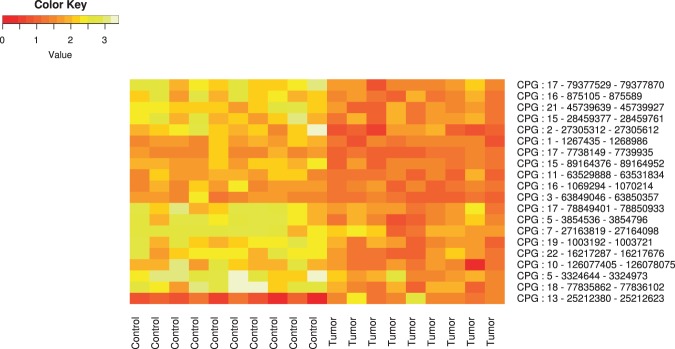
Colormaps for the CpG islands with highest difference of cleavage rates between normal and tumor tissues in meduloblastoma. X axis corresponds to samples, and Y axis, to CpG islands with their coordinates in the genome. The intensity of the color scale corresponds to the mean cleavage rate for CpG in every CpG island, which was calculated according to (1).

We intended to develop an approach that will make it possible to distinguish tumor tissue samples from normal ones by the dinucleotide cleavage characteristics. Our approach is based on the observation that methylated CpG islands have higher cleavage rates. Namely, the reads with the 5'-end nucleotide G preceded by C in genomic sequence will occur more frequently if C is methylated. For practical evaluation of this method, the data from EGA datasets was used (24 datasets of T-cell lymphoma, 15 high-coverage datasets of prostate cancer, big dataset (> 300 samples) of breast cancer, 24 datasets of hepatocarcinoma, and 37 datasets of medulloblastoma (average coverage ~ 40×) from ICGC database, as well as control datasets of healthy tissues). We selected a subset of EGA T-cell datasets with a good coverage (> 40×). Our approach allows the prediction of the difference between methylation statuses of CpG islands in healthy and tumor tissues. Figure 2 shows the CpG islands with the biggest absolute difference in their mean cleavage levels. In almost all cases we observed demethylation effect in the CpG islands of tumor tissues with one exception: the CpG island with coordinates chr13:-25,212,380–25,212,623, which could be associated with promoter region of the pseudogene TNFRSF1A. This gene is a well known oncogene involved in tumorogenesis^11^. We suggest that the unmethylated CpG island in the promoter region could lead to expression of the pseudogene thus promoting oncogenesis; however we have not validated this mechanism experimentally.

The detection of the cancer-specific methylation of particular CpG islands is a complicated task even in the presence of qualitative results of bisulfite-sequencing experiments. Therefore, for effective prediction of disease status from dinucleotide cleavage rates we employed some machine learning algorithms. At first, for each CpG island in each dataset (control and tumor) we computed an average CpG dinucleotide cleavage rate and turned every set into high dimension vector. Each element of this vector is an average CpG dinucleotide cleavage rate in a specific CpG island. These vectors were used for training of binary classifier on the basis of the support vector machine (SVM) method with linear kernel. This SVM classifier was used to predict the state of specific sample (cancer or normal). For training we used the same number of control and tumor datasets for each cancer type. In our research we applied SVM realization in e1071 package^12^ of R programming language. Corresponding script for samples classification based on cleavage rates of CpG islands and computation results are available on the GitHub page: https://github.com/LeonidU/DNA-Segmentation.

To control the accuracy of this algorithm, the jack-knife method was used. On each round of jack-knife we excluded random subset for each cancer type and trained the model on the remaining sets. Each subset contained an equal number of control and tumor sets. Then we made a prediction on the excluded subsets and computed true and false positive rates (Table 1).

**Table 1:** Results for of the prediction of tumor/normal status for different cancer types calculated by the SVM model described in the main text.

Cancer type	EGA ID	Percent of true positive cases	Percent of true negative cases	Percent of false positive cases	Percent of false negative cases
Breast Cancer	EGAD00001000126	79	60	21	40
Meduloblastoma	EGAD00001000816	76	96	24	4
T-Cell Lymphoma	EGAD00001002738	84	82	16	18
Prostate Cancer	EGAD00001000263	70	97	30	3
Hepatocarcinoma	EGAD00001001881	87	71	13	29

Percentage of positive cases and negative cases are calculated separately.

Our predictions were comparable with bisulfite sequencing based classifiers in the accuracy of prediction. The accuracy of the sample status prediction in^13^ was ~84–85%, which is comparable with our results.

## Discussion

We analyzed raw WGS data and found that cytosine methylation strongly affects the cleavage rate of CpG dinucleotides in the human genome. This fact agrees with recent observations of another group^3^. In all studied datasets from 1000 Genomes project the cleavage rate of methylated CpG dinucleotides was higher than the cleavage rate of other dinucleotides including unmethylated CpGs^1,2^. This observation was then used as a basis of the method that allows one to predict changes in the gene promoter epigenetics associated with cancer.

Using our method, one can estimate the total level of the whole-genome base methylation and compare the methylation degree in different genomic regions with similar functions at different stages of the organism development. The inferred correlation between the probability of mechanochemical cleavage of the DNA sugar-phosphate backbone and local nucleotide sequence enabled us to find an important connection between structural parameters of specific nucleotide sequences of DNA and their biological functions. Moreover, it should be possible to conduct a comparative analysis of methylation profiles for cells during differentiation and during aging by estimating the total DNA methylation level in various cells at least at the resolution of the CpG islands. The main problem for practical use of our method is a bad coverage of CpG islands and their heterogeneity in different cancer cells. For example, worst results of tumor/control separation were obtained for breast cancer with badly covered samples

On the basis of these results, the method can be developed for detecting the total level of the CpG methylation from the raw WGS data without any additional experiments. We hope that the developed method makes it possible to recognize base modifications in genomes other than methylation of cytosines in CpG dinucleotides^14^. There is a large space for further development of the designed method with regard both to the types of data analyzed and to the algorithm. Higher level of coverage of the genome could also increase a resolution of our predictions. Segmentation of DNA by sonication or other methods used in NGS provide tremendous amount of data concerning such an unusual mechanical property of DNA as the susceptibility to cleavage. Our approach opens up new possibilities for studies of both DNA physics and its relationships with double helix structure and epigenetics^15^.

## Methods

### Protocol of data processing

We used the whole genome sequencing samples of human individuals from the 1000 Genomes project^7^. The BAM files with WGS read alignments on a reference genome were downloaded from the FTP server of the 1000 Genomes project (ftp://ftp.1000genomes.ebi.ac.uk/vol1/ftp/phase3/data/). SAMtools suit was used to filter reads that only map on a forward strand of all autosomes of the human genome (excluding X and Y chromosomes) (*samtools view -f 35 -F 4 input.bam {1..22}> filtered.bam*)^15^. We also tested whether our filtration criteria affect average cleavage rates: we tested several other scenarios of filtration on 5 random datasets of the 1000 Genomes project. We checked if filtering out duplicated reads, usage of reads that only map on reverse strand, or filtering out the reads that map on CpG islands would affect average cleavage rates. The last filtering criteria were used in order to test if an increased cleavage rate of methylated CpG dinucleotides, as compared with that of unmethylated CpGs, can be explained by the difference in the CpG dinucleotide distribution in the genome. Most unmethylated CpGs are clustered in CpG islands; however, methylated CpGs can be observed both inside and outside the CpG islands. Thus, by filtering out the reads from CpG islands we compared the cleavage rates of methylated and unmethylated CpG dinucleotides from the rest of the genome. We found that the average dinucleotide cleavage rates do not depend on the filtering strategy, so we assumed that different distribution of methylated and unmethylated CpG dinucleotides in the genome did not affect our results substantially.

GRCh37 was used as a reference genome. To be sure about the type of dinucleotides observed we masked all low sequence complexity regions by RepeatMasker^16,17^. Thse frequency of XY dinucleotides was calculated not in the whole genome but in the 200-bp-long genome fragment around the read-start positions. By doing that we took into account all the sequence biases associated with read mapping positions^18^.

We used bisulfite-sequencing data from NGSmethDB database^19^ for the lymphoblastoid cell line samples (https://bioinfo2.ugr.es/NGSmethDB/methylation-maps/) to predict methylation status of cytosine in CpGs dinucleotides in the cell line that was used in the 1000 Genome project. We used only those CpG dinucleotides that were covered by more than 10 reads in bisulfite-sequencing data. We defined CpG dinucleotide as methylated or unmethylated if >90% of reads covering a specific dinucleotide contained C_M_ or C_U_ respectively at a corresponding position. CpG dinucleotides with some intermediate C_M_/C_U_ ratios were excluded from further analysis.

## Supplementary information

Supplementary Information.

## References

[1] Grokhovsky SL (2011). Sequence-specific ultrasonic cleavage of DNA. Biophys. J..

[2] Poptsova MS (2014). Non-random DNA fragmentation in next-generation sequencing. Sci. Rep..

[3] Garafutdinov Ravil R., Galimova Aizilya A., Sakhabutdinova Assol R. (2018). The influence of CpG (5′-d(CpG)-3′ dinucleotides) methylation on ultrasonic DNA fragmentation. Journal of Biomolecular Structure and Dynamics.

[4] Ziller MJ (2013). Charting a dynamic DNA methylation landscape of the human genome. Nature.

[5] Benjamini Y, Speed TP (2012). Summarizing and correcting the GC content bias in high-throughput sequencing. Nucleic Acids Res..

[6] Ehrlick M, Wang RYH (1981). 5-Methylcytosine in eukaryotic DNA. Science.

[7] 1000 Genomes Project Consortium (2015). A global reference for human genetic variation. Nature.

[8] Hackenberg M, Barturen G, Oliver JL (2011). NGSmethDB: A database for next-generation sequencing single-cytosine - resolution DNAmethylation data. Nucleic Acids Res..

[9] Esteller M (2002). CpG island hypermethylation and tumor suppressor genes: A booming present, a brighter future. Oncogene.

[10] Landan G (2012). Epigenetic polymorphism and the stochastic formation of differentially methylated regions in normal and cancerous tissues. Nat. Genet..

[11] Egusquiaguirre SP (2018). The STAT3 target gene TNFRSF1A modulates the NF-κB pathway in breast Cancer cells. Neoplasia..

[12] Dimitriadou E (2008). Misc functions of the Department of Statistics (e1071). R package..

[13] Yegnasubramanian S (2004). Hypermethylation of CpG Islands in Primary and Metastatic Human Prostate Cancer. Cancer Res..

[14] Semyonov D., Nechipurenko Y. Non-Canonical GC Base Pairs and Mechanochemical Cleavage of DNA https://arxiv.org/abs/2001.03561 (2009). 10.1002/bies.202000051 32830350

[15] Nechipurenko DI (2014). Modeling of mechanochemical DNA cleavage by action of ultrasound. Biofizika..

[16] Li H (2011). A statistical framework for SNP calling, mutation discovery, association mapping and population genetical parameter estimation from sequencing data. Bioinformatics.

[17] Smit, A., Hubley, R. & Green, P. RepeatMasker Open-4.0.6 2013-2015. Available at, http://www.repeatmasker.org. (Accessed: 20th January 2019).

[18] Il’icheva I (2016). Structural features of DNA that determine RNA polymerase II core promoter. BMC genomics.

[19] Lebrón R (2017). NGSmethDB 2017: Enhanced methylomes and differential methylation. Nucleic Acids Res..

