# Supplementary material for: A Method for Identification of the Methylation Level of CpG Islands From NGS Data
Source: Sci Rep. 2020 May 25;10:8635. doi: 10.1038/s41598-020-65406-1 (PMC7248081; doi:10.1038/s41598-020-65406-1)
Supplement: Supplementary file 1 — Supplementary Information. [file 41598_2020_65406_MOESM1_ESM.pdf]

A Method for Identification of the Methylation Level of CpG Islands From NGS Data

Supplementary Materials

Leonid A. Uroshlev*
1. Engelhardt Institute of Molecular Biology, Russian Academy of Sciences, Moscow, Russia
2. Vavilov Institute of General Genetics, Russian Academy of Sciences, Moscow, Russia
leoniduroshlev@gmail.com
Eldar T. Abdullaev
3. Max Planck Institute for Molecular Genetics, Berlin, Germany
abd.eldar1993@gmail.com
Iren R. Umarova
4. Faculty of Computational Mathematics and Cybernetics, Moscow State University, Moscow, Russia
umarovairen@gmail.com
Irina A. Il'icheva
1. Engelhardt Institute of Molecular Biology, Russian Academy of Sciences, Moscow, Russia
imb_irina@rambler.ru
Larisa A. Panchenko
4. Faculty of Biology, Moscow State University, Moscow, Russia
larandr@mail.ru
Robert V. Polozov
5. Institute of Theoretical and Experimental Biophysics, Russian Academy of Sciences, Puschino, Russia
polrob@mail.ru
Fyodor A. Kondrashov
6. Institute of Science and Technology Austria, Klosterneuburg, Austria
fyodor.kondrashov@ist.ac.at
Y**ury D. Nechipurenko***
1. Engelhardt Institute of Molecular Biology, Russian Academy of Sciences, Moscow, Russia
yurii.nechiporenko@gmail.com
Sergei L. Grokhovsky
1. Engelhardt Institute of Molecular Biology, Russian Academy of Sciences, Moscow, Russia

Table 1 Suppl. *Average cleavage rates for methylated and unmethylated CpG dinucleotides.*

Methylation status	cleavage rate	number
C_MG (methylated C)	1.95	199554
C_UG (unmethylated C)	1.49	16596

Notes. *The number of observed CpG dinucleotides with known epigenetic status is shown in the third column.*

Table 2 Suppl. *Duncan test for sample mean.*

	Duncan test; Homogenous Groups, alpha = ,05000											
Cell No.	NN	Y Mean	1	2	3	4	5	6	7	8	9	10
3	AG	0.78	****									
11	GG	0.81	****									
4	AT	0.90		****								
16	TG	0.93		****								
2	AC	0.93		****								
12	GT	0.94		****								
17	TT	1.00			****							
15	TC	1.00			****							
10	GC	1.01			****							
1	AA	1.01			****							
9	GA	1.03			****	****						
6	CC	1.06				****	****					
8	CT	1.09					****	****				
14	TA	1.12						****				
5	CA	1.23							****			
18	C_UG	1.49								****		
7	CG	1.84									****	
13	C_MG	1.95										****

Table 3 Suppl. *P-values for nonparametric Kruskal – Wallis test for dinucleotides: CG, C_MG, and C_UG with alpha=0.05.*

Group	CG	C_MG	C_UG
CG		0.053	<1e-10
C_MG	0.053		<1e-10
C_UG	<1e-10	<1e-10	

Table 4 Suppl. *Mean values of the relative cleavage rates for dinucleotides, standard deviations and 95% confidence limits for the population mean.*

Dinucleotide	Mean	SD	The 95% confidence limits	
			Lower limit	Upper limit
AA	1.012	0.073	0.998	1.027
AC	0.928	0.077	0.912	0.943
AG	0.776	0.094	0.757	0.794
AT	0.902	0.093	0.884	0.921
CA	1.225	0.098	1.206	1.245
CC	1.056	0.092	1.038	1.075
CT	1.093	0.240	1.070	1.116
GA	1.028	0.117	1.014	1.042
GC	1.008	0.070	0.979	1.038
GG	0.809	0.150	0.781	0.837
GT	0.940	0.140	0.920	0.960
TA	1.119	0.102	1.101	1.137
TC	1.003	0.269	0.989	1.017
TG	0.926	0.089	0.903	0.949
TT	0.997	0.071	0.978	1.017
CG	1.845	0.114	1.797	1.892
C_MG	1.950	0.097	1.897	2.003
C_UG	1.489	0.239	1.442	1.537

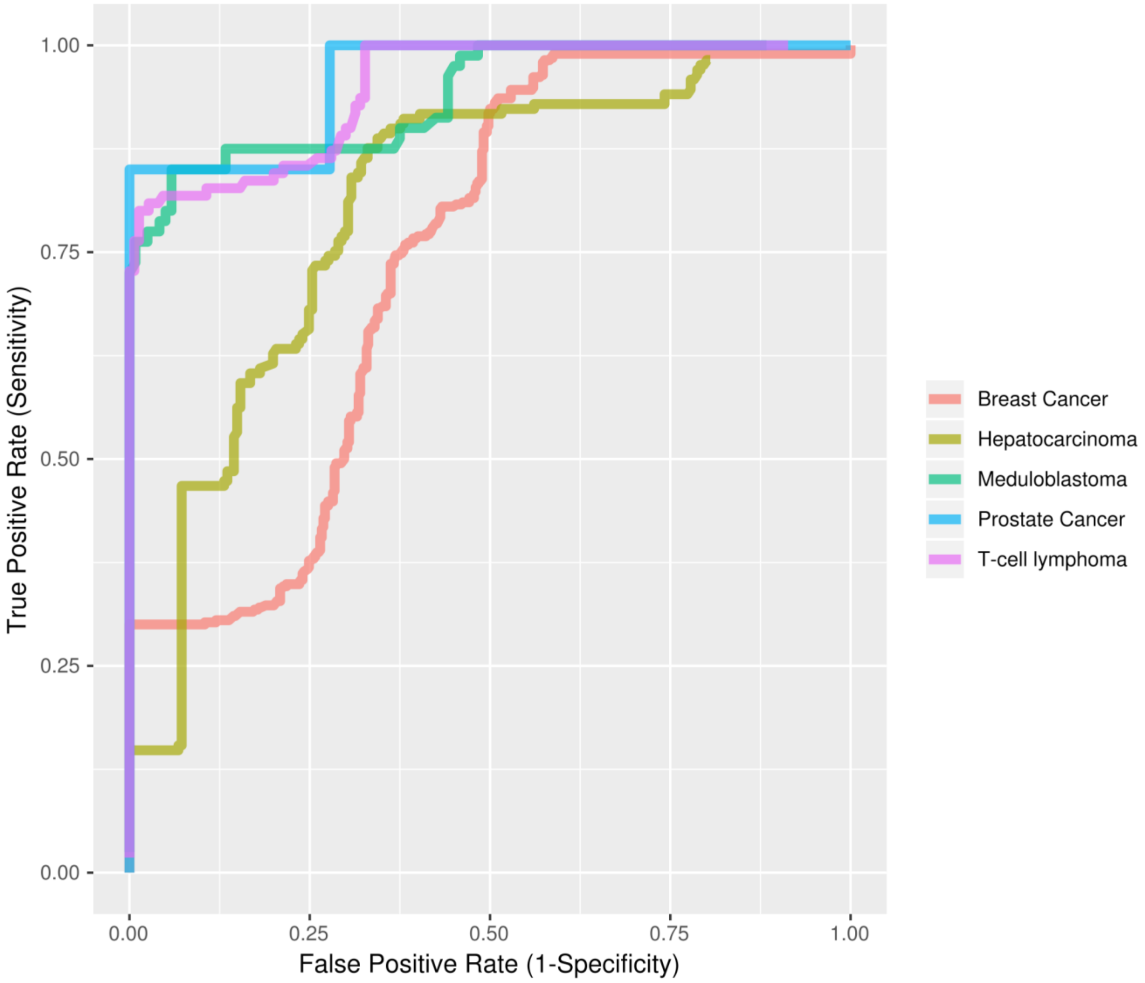

Fig. 1 Suppl. *ROC-curves of SVM-classificator for different types of cancers*
